# Partnerships for promoting dissemination of mental health research globally

**DOI:** 10.4103/0019-5545.69207

**Published:** 2010-01

**Authors:** Helen Herrman

**Affiliations:** Secretary for Publications, World Psychiatric Association and Orygen Youth Health Research Centre, The University of Melbourne, Australia

**Keywords:** Psychiatric publications, psychiatric research, low- and middle-income countries, indexation

## Abstract

Low-and middle-income countries (LAMIC), with more than 80% of the global population, bear the greatest burden of mental disorders. Yet these countries are very under-represented in published psychiatric research. There are barriers to publication of mental health research from LAMIC and to the representation of research from these countries in the main literature databases worldwide. The World Psychiatric Association (WPA) aimed to help investigate the reasons for the under-representation of LAMIC in published psychiatric research and consider how to support improved research dissemination, relevant for better mental health and mental health care in all countries. As part of its work plan for the years 2008 to 2011, WPA developed a project to encourage efforts to offer support to psychiatric journals in LAMIC. A WPA publications taskforce was appointed in 2008 to promote the dissemination of research from LAMIC. The taskforce began work together with journal editors to strengthen their chances of being indexed in international databases. Among the first journals participating in the project was the IJP, which is now an inspiration and source of support for other journals.

When Prof. T. S. Sathyanarayana Rao, the editor of the Journal, announced the news last year that the National Library of Medicine accepted the Indian Journal of Psychiatry (IJP) for indexation in Medline, he described it as a long cherished dream. It was a dream shared by many. Dr. Rao expressed his gratitude to all the supporters of the IPS and at the Journal. The journal he thanked the editorial team, the editorial board members, the journal committee, Indian and International Advisers, the reviewers and authors, and the publication house. He also thanked the World Psychiatric Association (WPA) and its taskforce for research dissemination for support.

Indeed the excellent news of the Journal’s indexation in this major database brings to fruition a dream, or rather a vision, shared by the World Psychiatric Association (WPA). The WPA exists to promote the advancement of psychiatry and mental health for all peoples of the world (www.wpanet.org). Its aims to include and increase the knowledge and skills necessary for study in the field of mental health and in the care of people with mental illnesses, as well as preserve the rights of people with mental illnesses, and promote the development and observance of the highest quality and ethical standards in psychiatric care, teaching, and research. As part of its work plan for the years 2008 to 2011, defined by the President, Prof. Mario Maj,[1,2] WPA has developed a project to encourage efforts to offer support to psychiatric journals in the low- and middle-income countries (LAMIC). A WPA publications taskforce was appointed in 2008, to promote the dissemination of research from LAMIC. The taskforce began the work together with the journal’s editors, to improve the quality of their publication and to strengthen their chances of being indexed in international databases. Among the first journals participating in the project was the IJP, which is now an inspiration and source of support for other journals.

## Need for support for research dissemination in low- and middle-income countries (LAMIC)

The WPA joins other major organizations in advocating for wider dissemination of research findings that contribute to better mental health and mental healthcare in all countries.[3] Publishing local research information in local scientific journals has an important influence on policy and practice at the national and regional levels and encourages the expansion of cost-effective and culturally appropriate health services. International and multicultural psychiatric research is also required to enhance the understanding of the determinants of mental health and the options for the prevention and treatment of illnesses.[4,5] Where the needs are greatest, however, the least research is available or accessible. LAMIC, with more than 80% of the global population, bear the greatest burden of mental disorders. Yet there is a striking under-representation of these countries in the published psychiatric research.[4–6] This situation reflects the limitations in the production of scientific research. It also reflects barriers to the publication of mental health research in LAMIC and to the representation of research from these countries in the main literature databases worldwide.

The level of submission from LAMIC in high-impact indexed journals is less than 20%[7] and the proportion of articles published is even lower:[8] A search in the Institute of Scientific Information (ISI) Web of Science database from 1992 to 2001[3] has reported that LAMIC (n=152) contributed to only 6% of the international mental health research. A recent review of all original contributions from 2002 to 2004, in the six highest impact factor journals, in the field of psychiatry revealed that only 3.7% of the published articles were submitted by authors from LAMIC.[9] Moreover, a survey in the editorial and advisory boards of ten leading psychiatry journals showed a low representation of LAMIC.[10]

A further major obstacle to disseminating LAMIC research is the scarcity of indexed journals with a strong LAMIC focus, for example, journals published in LAMIC.[3] Approaches to addressing this problem include the development of new regional journals, for example, the Asian Journal of Psychiatry and Asia Pacific Psychiatry, and the publication of major international journals with a focus on or special interest in LAMIC. Another significant development is the publication of World Psychiatry, the official journal of WPA. Under the editorial leadership of Prof. Mario Maj it has developed over the past 10 years to have an impact factor greater than four, among the top 10 clinical psychiatry journals worldwide. However, an important additional approach to solving this problem is to increase the profile of high quality national psychiatric journals already published. Barriers to the indexation of journals appear to contribute to the difficulties in achieving a fair representation in the main literature databases for the science produced in LAMIC.[4]

Local initiatives to develop information networks between researchers and mental health professionals are evident in some countries. With the increasing number of published journals, however, retrieving the best information has become increasingly difficult and requires researchers to use indexation databases. The use of indexation databases is now an equally important requirement for any publication that pursues adequate visibility and impact of research published in the journal. Two of the most relevant indexation systems for psychiatric journals are Medline / PubMed, a bibliographic database developed by the US National Library of Medicine, and the citation indexes of the ISI (Institute for Scientific Information), now part of Thomson Scientific, available online under the name Web of Science.

## WPA taskforce on research dissemination in LAMIC

The WPA decided to investigate the reasons for the under-representation of LAMIC in published psychiatric research and to consider how to support improved research dissemination. The decision was inspired by the earlier work from the World Health Organization (WHO)[11] and by the work of a small number of journal editors in LAMIC, including The Revista Brasileira de Psiquiatria (RBP), which was one of the first LAMIC publications to be indexed in the major databases. Recognizing that a major national and Asian journal such as the IJP remained outside the main international databases also inspired it.

The WPA’s publications committee began by conducting a survey in Medline and ISI Web of Science in order to identify the global distribution of journals in the field of psychiatry. It found 222 indexed psychiatric journals. Of these, 213 originated from high-income countries and only nine (4.1%) were from the middle-income countries: five from the European region, where there were two existing psychiatric journals indexed in both ISI / Medline databases, and three others indexed in Medline; and four from Latin America, with one journal indexed in each database and two journals present in both. None came from the low-income countries and none were identified in the Asian or African regions. The investigators also obtained information about unindexed journals from the WPA zonal representatives and from a project of the World Forum for Global Research, as also from the World Bank. Together, these sources identified 118 LAMIC psychiatric journals not indexed in Medline or ISI, attesting to the activity of researchers and readers globally.[6] The committee’s next activity, based on the surveys and led by Dr. Christian Kieling, was to establish a directory of all available mental health and psychiatric journals worldwide and link it to the WPA website. The WPA had 130 member societies in 110 countries, and with the help of its zonal representatives it worked to maintain and develop the directory as a basis for advocacy and action.

The search in Medline and the ISI did not reveal any psychiatric journal indexed from the Asian region in either of these two databases. For example, despite the long history of the IJP, it was not then indexed in either of these databases. The taskforce, through its members, and Prof. Vikram Patel in particular, became aware of the long and distinguished history of the IJP, and entered into communication with the editor Dr. TSS Rao. Dr. Rao, as an interested and active participant, emphasized a number of features with relevance to the success in application for indexation.[4]

The IJP promotes original research in psychiatry and behavioral sciences. It is in the forefront of the mental health movement in India. It has remained in circulation for more than 50 years and the readership has grown continuously. IJP is possibly the oldest psychiatric journal in Asia and is the primary mental health academic journal for one-sixth of humanity. The journal provides immediate and free access to all published articles. It does not charge the authors for submission, processing or publication of the articles. The IJP is a journal comprising of good quality articles, and a broad international board. The number of issues is four per year, and the number of original articles around five per issue. The rejection rate is 23% and the length of time for publication is 162 days. In 2008 the journal was indexed in the following databases: (a) SCOPUS; (b) DOAJ; (c) Index Copernicus; (d) Health and Wellness Research Center; (e) Health Reference Center Academic; (f) InfoTrac One File; (g) Expanded Academic ASAP; (h) Genamics Journal Seek; (i) Ulrich’s International Periodical Directory; (j) EBSCO Publishing’s Electronic Databases; and (k) Google Scholar. The taskforce considered that in terms of content quality, the Journal was ready for submissions in both Medline and ISI, and invited Dr. Rao to the taskforce’s first workshop with editors at the World Congress of Psychiatry in Prague, in September 2008. The taskforce also noted the importance of a stable and supporting relationship with its professional society, the IPS.

In preparation for the 2008 workshop, colleagues from WPA and the Global Mental Health Movement led by Prof. Jair de Jesus Mari helped to contact editors from LAMIC in Africa, Asia, Eastern Europe, and Latin America, to identify potential journals to target for indexation (Medline and ISI). The task force appraised the quality of 26 non-indexed journals, whose editors were interested in participating, and invited eight editors to attend, two each from the four WPA regions. The quality criteria included: affiliation to a professional mental health society; regular publication of at least four issues per year over the past few years; comprehensive national and international editorial boards; publication of original articles, or at least abstracts, in English; some level of current indexation; evidence of a good balance between original and review articles in publications; and a friendly access website. Prof. Mari, former editor of RBP and Dr. Kieling, current associate editor, described the experience of RBP in improving the journal on each of these criteria, and the other editors described the work of their journals. The discussion established an atmosphere of peer support. This support continues, with the taskforce offering continued advice through email and other forms of contact.

The taskforce now aims to offer concentrated support for those selected journals, and to assist all LAMIC mental health editors in improving the quality of their journals and fulfilling the requirements for full indexation. Several journals, including some whose editors have worked with the WPA project, have achieved indexation in the past two years. The survey was repeated in 2009. Four publications were added to the databases: one from Brazil, two from South Africa, and one from Turkey. Three other journals that were indexed only in Medline two years ago are now part of the Web of Science database: one each from Croatia, Poland, and Turkey. Despite these inclusions, the proportion of journals from LAMIC remains virtually unchanged (13 of 235 [5·5%]).[5] Subsequently, the IJP was the first journal from Asia to achieve indexing in Medline.

The next step is to scale up the support for journal editors in the LAMIC. The work of the IJP can provide inspiration and peer support within a framework, as established by the WPA taskforce. The work can be monitored through the database and the network of editors. The work of this taskforce will continue with workshops at major WPA meetings, to include new editors as well as those who can describe their success stories. We also hope to establish editorial fellowships for junior editors in the office of high quality journals. The IJP and other journals with a record in achieving high standards, together with the funding agencies, can partner with journals from LAMIC to improve their quality and strengthen their chances of being indexed.

At present, we look forward to a continued collaboration so that others can learn from the experience of the IJP and achieve indexing in Medline and eventually in ISI. The IJP’s achievement may serve as an example for other LAMIC journals to pursue indexation in major databases, as a strategy to widen the international foundation of psychiatric research. There is an important need for the inclusion of LAMIC psychiatric publications in the major indexation databases. This process will require multiple agents to partner with journals from LAMIC, to improve their quality and strengthen their chances of being indexed.
